# Comparative analysis of diversity and utilization of edible plants in arid and semi-arid areas in Benin

**DOI:** 10.1186/1746-4269-10-80

**Published:** 2014-12-23

**Authors:** Alcade C Segnon, Enoch G Achigan-Dako

**Affiliations:** Horticulture and Genetics Unit, Faculty of Agronomic Sciences, University of Abomey-Calavi, 01 BP 526, Cotonou, Republic of Benin

**Keywords:** Benin, Dry areas, Edible food plants, Food security, Species richness, Socio-cultural attributes, Farming practices

## Abstract

**Background:**

Agrobiodiversity is said to contribute to the sustainability of agricultural systems and food security. However, how this is achieved especially in smallholder farming systems in arid and semi-arid areas is rarely documented. In this study, we explored two contrasting regions in Benin to investigate how agroecological and socioeconomic contexts shape the diversity and utilization of edible plants in these regions.

**Methods:**

Data were collected through focus group discussions in 12 villages with four in Bassila (semi-arid Sudano-Guinean region) and eight in Boukoumbé (arid Sudanian region). Semi-structured interviews were carried out with 180 farmers (90 in each region). Species richness and Shannon-Wiener diversity index were estimated based on presence-absence data obtained from the focus group discussions using species accumulation curves.

**Results:**

Our results indicated that 115 species belonging to 48 families and 92 genera were used to address food security. Overall, wild species represent 61% of edible plants collected (60% in the semi-arid area and 54% in the arid area). About 25% of wild edible plants were under domestication. Edible species richness and diversity in the semi-arid area were significantly higher than in the arid area. However, farmers in the arid area have developed advanced resource-conserving practices compared to their counterparts in the semi-arid area where slash-and-burn cultivation is still ongoing, resulting in natural resources degradation and loss of biodiversity. There is no significant difference between the two areas for cultivated species richness. The interplay of socio-cultural attributes and agroecological conditions explains the diversity of food plants selected by communities.

**Conclusions:**

We conclude that if food security has to be addressed, the production and consumption policies must be re-oriented toward the recognition of the place of wild edible plants. For this to happen we suggest a number of policy and strategic decisions as well as research and development actions such as a thorough documentation of wild edible plants and their contribution to household diet, promotion of the ‘’bringing into cultivation” practices, strengthening of livestock-crop integration.

## Background

Agrobiodiversity is understood as “*the variety and variability of living organisms that contribute to food and agriculture in the broadest sense, and that are associated with cultivating crops and rearing animals within ecological complexes*” [1, 2]. A sustainable utilization of agrobiodiversity and associated ecosystem services through diversified farming systems is advocated to be a robust approach for addressing food security and the sustainability of agricultural systems [3–8]. However, how agrobiodiversity is used to address food security and sustainability of agricultural systems in smallholder farming systems in arid and semi-arid areas of West Africa needs to be investigated and documented so as to inform policy decisions. In fact, the challenge of providing sufficient food for the increasing population while preserving natural resources is higher in arid and semi-arid areas [9, 10]. In these areas, agricultural production systems are faced not only with persistent water scarcity and frequent drought, but also with high climatic variability, land degradation, desertification, and widespread poverty [9]. The strong climatic variations and irregular rainfalls that characterized agro-ecosystems in dry areas make harvest of staple and cash crops highly uncertain, especially in West African Sahel and dry savannas [11]. These constraints are expected to intensify as a result of population growth, urbanization and climate change, which will likely exacerbate food insecurity in these areas, that are already vulnerable to hunger and under-nutrition [12]. In this context, increased knowledge of the functionalities of agrobiodiversity will help build the social and natural science evidence-base to allow formulation of adequate intensification strategies [13]. These context- and location-specific strategies require a clear understanding of food production and consumption systems.

Previous investigations in West Africa explored the relationships that communities have developed with their environment and surrounding biological resources, including the utilization of plant resources. From those previous findings, we understood that the value and utilization of plant resources in communities are influenced by sociolinguistic membership [14–22] and to some extent geographical contexts [23, 24]. However, gap still exists in the understanding of how ecological and socioeconomic contexts shape the utilization of agrobiodiversity and its contribution to food security in this region. Most studies focus on the consumption and variation of knowledge of single species (e.g. *Parkia biglobosa* [14], *Sclerocarya birrea* [15], *Tamarindus indica* [22, 25], *Blighia sapida* [16], *Adansonia digitata* [26]) or categories of species (e.g. woody species [27, 28], Non-Timber Forest Products species [20, 29], vegetables species [24, 30]) and the linkage between agrobiodiversity, food consumption and security was partially addressed. Food security is a complex condition with four key dimensions namely food availability, food utilization, food accessibility and food system stability [31]. Understanding how food security is achieved in arid and semi arid areas while sustaining the use of agrobiodiversity will certainly provide insight into plant resources preservations mechanisms, food production strategies, and sustainable livelihoods.

The objectives of this study are to assess the diversity and utilization of edible plant resources, and analyze farming practices in relation to agroecological contexts in agricultural communities of two contrasting regions in Benin. The following questions were addressed in this paper: (1) What are the edible plants used in the arid and the semi-arid regions of Benin and what is the place of wild resources? (2) Is the diversity of edible plants used in the semi-arid and arid regions the same? (3) Are ecological differences between the arid versus semi-arid regions reflected in differences in farming practices and use of edible plant resources? (4) Are edible plants’ choice and utilization similar in communities of the arid and the semi-arid regions? We hypothesized that the use of edible plant resources (wild and cultivated) is affected by agroecological conditions, sociolinguistic attributes and farming practices of rural communities.

## Methods

### Study area

**Figure 1:**
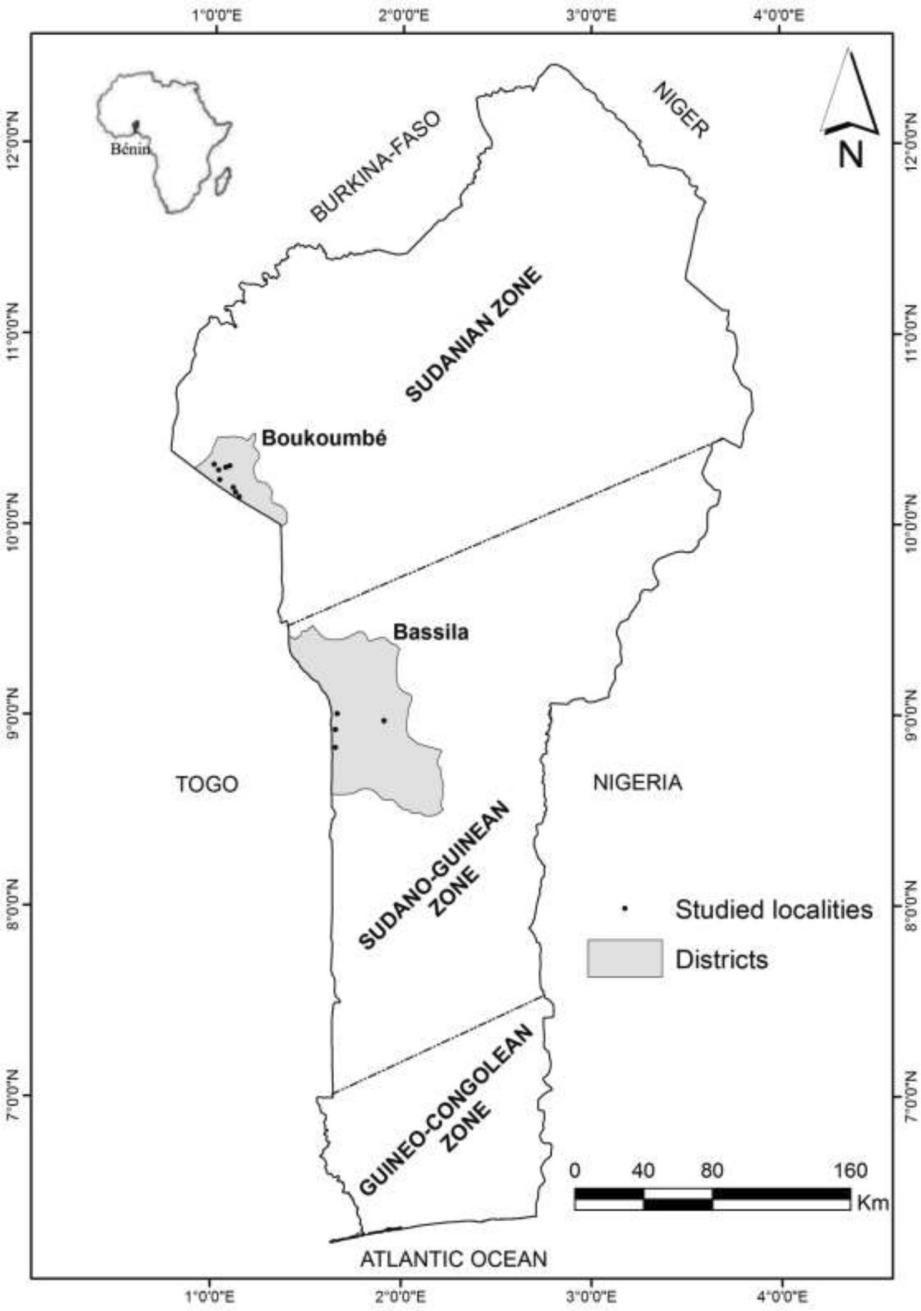
**Location of the study areas.**

This study was conducted in two ecological regions of Benin (West Africa) namely the Sudanian and the Sudano-Guinean regions. Benin is located on the Atlantic coast, and borders Nigeria to the east, Togo to the west, and Burkina Faso to the northwest, and Niger to the north (Figure 1). The vegetation pattern shows a humidity gradient northward as a result of the joint effects of the climate and the soils [32].

The Sudanian region is a woodland and savanna region with ferruginous soils. The rainfall is unimodal with a mean annual for about 1000 mm (Table 1). The temperature ranges from 24 to 31°C [32, 33]. The main sociolinguistic groups are Bariba, Fulani and Otamari and related sociolinguistic groups [34]. Farming systems are mainly based on cotton and cereal cultivation and livestock breeding. In the Western part, farming systems are limited by both land degradation and availability leading to population migration into central part of the country [35].

**Table 1:** **Bio-geographical and socioeconomic characteristics of the study areas**

	Semi-arid zone (Bassila)	Arid zone (Boukoumbé)
Biophysical gradient	Sudano-Guinean (7°30-9°30 N) region	Sudanian region (9°30-12° N) region
Annual rainfall	1100 – 1300 mm	900 – 1100 mm
Active vegetation period	200 days	145 days
Relative Humidity		
Min	30 – 70%	<30%
Max	<80%	45 – 75%
Temperature	25 to 29°C	24 to 31°C
Forest cover	50% of the total area of the municipality of Bassila is covered by forest reserves [Monts Kouffè Forest Reserve (201 000 ha), Pénéssoulou Forest Reserve (5 470 ha), Bassila Forest Reserve (3 320 ha) and Wari Maro Forest Reserve (1/3 of 107 500 ha)]	Not available
Farming systems	Cereal- (maize and sorghum) and Yam-based	Cereal-based (sorghum and pearl millet)
Socioeconomic gradient		
Food insecurity	Moderate food insecurity	Severe food insecurity
Poverty incidence	Low	High
	Less than 40% of poor household	72% of poor household
	20% of population living in extreme poverty	51% of population living in extreme poverty
Number of villages surveyed in each municipality	04	08
Sociolinguistic groups surveyed (number of villages) in each municipality	Ditamari (01), M’Bermé (01), Nagot (01), Lokpa (01)	Ditamari (06), M’Bermé (02)
Number of respondents	90	90

Data assembled from Adomou [32], Akoègninou *et al.* [33], Bongi *et al.* [38], MAEP [39].

The Sudano-Guinean region is a transitional zone between the Guinean forests in the south and the Sudanian woodlands and savannas in the north and is characterized by a vegetation mosaic of forest islands, gallery forests, and savannas. The rainfall is unimodal and lasts for about 200 days with an annual mean rainfall varying from 1100 to 1300 mm (Table 1). The temperature varies from 25 to 29°C [32, 33]. The main sociolinguistic groups are Fon, Yoruba-Nagot and related sociolinguistic groups [34]. Other ethnic groups found include Otamari, Yom-Lokpa and related sociolinguistic ethnics groups. These ethnic groups represent two sociolinguistic groups that form the principal actors of migratory dynamics in Benin [23, 36]. Members of these groups are motivated to leave their homes in the hilly and over-populated North-West part to the central part in search of the virgin and fertile lands [23, 35–37]. Fulani herders are also found in the Sudano-Guinean region because of their nomadic pastoralist lifestyle. The yam-based cropping systems are dominant [35] while rice cultivation is also important. Cotton and cashew nut are the major cash crops in the Sudano-Guinean zone.

Based on biophysical and socio-economic gradients [32, 33, 38, 39], we selected Boukoumbé and Bassila municipalities for field investigations (Table 1, Figure 1). Boukoumbé (10°10'36.1"N and 01°06'22.0"E) is located in Atacora department (north-western Benin) and belongs to the arid Sudanian region. Main ethnic groups in Boukoumbé include Ditamari, M’Berme, Natimba and Berba, which form the Otamari socilolinguistic group [34]. Otammari ethnic groups compose 92.4% of the population of Boukoumbé [34]. Traditional social system of the Otammari is based on crop production [36, 40]. Limited access to arable land due to the Atakora mountain chain and land degradation lead Otammari people to leave their homes to the central part of the country [23, 35–37]. The total population of Boukoumbé is 60568 with a population density of 58 habitants per km^2^ [41]. The total land area is 1036 km^2^. Eighty six percent of household depend primarily on agriculture for their livelihood [41]. About 72% of households in the department of Atacora (in which belongs Boukoumbé) are poor (the highest proportion at national level) and 51% of its population live in extreme poverty [38]. To reduce this severe food insecurity an emergency programme of the Benin government was implemented from 2009 to 2011 through various emergency projects [39]. Bassila (09°01'00.1"N and 01°40'00.1"E) is located in Donga department of (upper central Benin) and belongs to the semi-arid Sudano-Guinean region. Main ethnic groups in Bassila include in order of importance Nagot, Anii and Kotokoli ethnic groups [34]. Nagot people are considered as native dwellers of Bassila although they have known to be originated from Yoruba people of Nigeria with whom they share indeed many similarities [23]. Anii and Kotokoli originated from Togo. Other ethnic groups such Otamari, Lokpa and Fulani are found in Bassila due to population migration. Indeed, Bassila as well as other municipalities in the transitional Sudano-Guinean zone between the Sudanian zone in the north and the Guinean zone in the south, is a receptacle of strong dynamic migration [23, 36]. Otamari are from the Atakora mountain chain region while Lokpa people are from municipality of Ouaké (in northern Bassila) [34]. Apart Fulani who has a nomadic pastoralist lifestyle, other ethnic groups in Bassila are mainly tillers [34, 40]. The total population of Bassila is 71511 with a population density of 13 habitants per km^2^ [41]. The total land area is 5661 km^2^. Currently, Bassila is the second largest municipality of Benin and about half of its total land area is covered with forests. Eighty three percent of household depend primarily on agriculture for their livelihood [41]. About 40% of households in the department of Donga (in which belongs Bassila) are poor with 20% of its population living in extreme poverty [38]. Also, Bassila is in a moderate but steady food insecurity situation [39].

### Data collection

In each municipality, we identified main agricultural production zones with local extension service agents. Afterwards, villages were randomly selected in these zones and the number of villages by municipality is proportional (11 to 12%) to the total number of villages that each municipality holds. Bassila holds 31 villages whereas Boukoumbé holds 71 villages [41]. We surveyed four villages in Bassila (Adjiro, Aoro-Lokpa, Camp pionier and Mondogui) and eight villages in Boukoumbé (Dimatema, Dipokor 1, Ditchendia, Koukongou, Kounadogou, Koutchata, Okouaro and Tassayota). Each surveyed village exhibit a dominant sociolinguistic group. A sociolinguistic group is understood here as a group in which a member inherits a common language of communication and shares social attributes such as customs, history, and food habits as recognized by Achigan-Dako *et al.* [24]. We carried out a focus group discussion in each village with about 20 community members and with a balanced representation of men, women, and different age groups. The socially defined age classes (youth - an unmarried individual; an adult - an individual married, but not considered an elder; and elder [42]) were considered. Participants in focus group discussions are community’s members locally recognized as knowledgeable about edible plants. Those participants were invited by the chief of the village and their peers. We obtained a permission of the chief of each village before conducting a focus group discussion, and followed the ethical guidelines of the International Society of Ethnobiology [43]. Participants were asked to build a free and an agreed list of edible plants consumed in the villages and to indicate their status (e.g. cultivated, wild or under domestication), utilizations and plant parts used. For local people, a “cultivated” species is a crop plant that is only known to be cultivated in the village; a “wild” species referred to any other food plants ranging from truly wild (entirely wild and collected only when needed) to wild-protected (maintained and protected or preserved in fields and fallows or around habitats with a sort of ownership), and semi-domesticated plants (cultivated in home gardens or in selected parts of cultivated fields where farmers tend to conduct diverse experiments); an “under domestication” species referred to plants of “wild” category that are not considered fully cultivated yet, and are no longer considered truly wild. It includes wild-protected and semi-domesticated plants, and species that are cited as cultivated by some participants and as wild by other during focus group discussion. Cultivated plants that grow spontaneously in the bush or fallow were not considered as wild species. At the end of each focus group, a guided tour was organized in the village to collect vouchers of edible plants previously listed (by their local names) by participants. The vouchers were selected with the help of two informants who were consensually selected among the participants. Species were taxonomically identified following Akoègninou *et al.* [33]. We also used the illustrated reference book of traditional vegetable species in Benin [44] and an illustrated reference book of trees, shrubs and lianas of dry zones of West Africa [45] to identify plant species. Voucher specimens were sent to the National Herbarium of Benin at University of Abomey-Calavi for further taxonomic confirmation.

In addition to focus group discussions, we carried out semi-structured individual interviews with 90 farmers in each municipality using a questionnaire. Participants were randomly selected and included in the survey after obtaining their verbal prior informed consent. Participant included is an individual who has his farm and hold the decision-making of activities to be implemented on it. Farmers were asked to describe their farming practices starting from land clearing and preparation until post-harvest practices on field.

### Data analysis

To estimate edible plant species richness in the two municipalities, we translated the utilization patterns of village communities into presence-absence data to generate species accumulation curve using EstimateS statistical device [46]. The species accumulation curve represents the number of edible plant species as a function of some measure of the sampling effort employed in the surveys. It yields the asymptotic richness of the assemblage and the computation of the asymmetrical confidence interval of the estimates [46, 47]. To assess the diversity of edible plant species in the two municipalities, we used the Shannon-Wiener index computed in EstimateS based on the species accumulation curve [46].

To compare the estimated edible plants species’ richness and diversity between the two areas and between cultivated and wild groups, we performed a *t*-test, a Welch’s test or a two-sample Wilcoxon test when appropriate. We used *t*-test when normality and homoscedasticity assumptions were met, Welch’s test when normality assumptions was met but not homoscedasticity and two-sample Wilcoxon test when normality and homoscedasticity assumptions were not met [48]. Shapiro-Wilk’s test for normality and Levene’s test for equality of error variances were used to test the normality and the homoscedasticity assumptions respectively. We also used a range of descriptive statistics to account for species diversity and use patterns.

To test if farming practices used by farmers were independent of their agroecological region, we used Fischer’s exact test or chi-square test when appropriate. Fischer’s exact test was used when some cells of the contingency table had frequencies less than five and these cells represent more than 20% of the total number of cell in the contingency table [48, 49].

To assess relationships among village communities in term of the utilization of edible plants species, we performed a hierarchical cluster analysis based solely on cultivated species, on wild species and then wild and cultivated together. Dendrograms were obtained using the complete linkage algorithm based on Jaccard coefficient of similarity [50] generated from the presence–absence data matrix in which villages were considered as sampling units and species as variables and scored, for each village, as 1 when present or 0 if not. All statistical analyses were performed using R version 3.0.2 [51].

## Results

### Diversity and richness of edible plants species and farming context in semi-arid and arid regions

**Figure 2:**
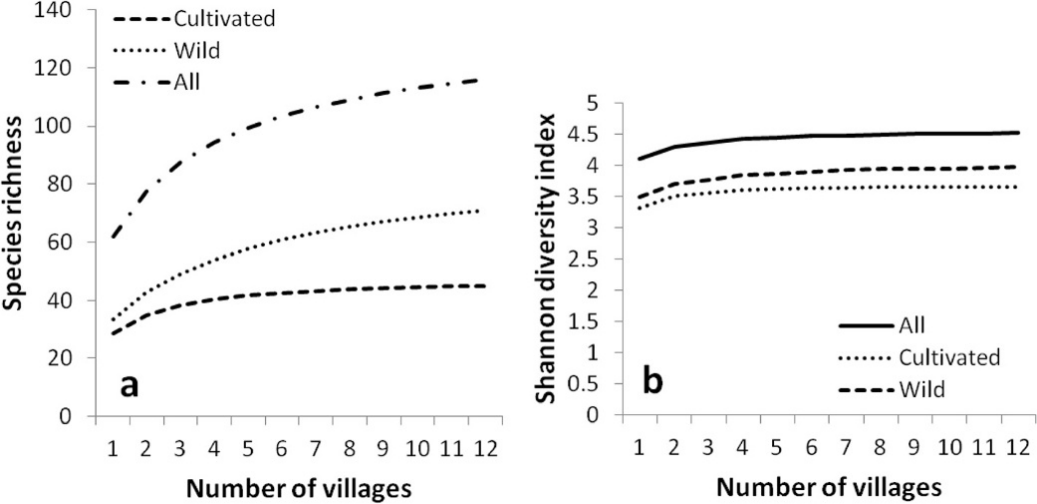
**Estimated species richness (a) and Shannon diversity (b) for the edible food plant species in two arid (Boukoumbé) and semi-arid (Bassila) areas of Benin based on incidence data.**

The total edible plant species richness was estimated to vary between 110 and 122 with an absolute value of 115 species (Figure 2a). Species belong to 48 families and 92 genera. Families with the highest genera and species richness include Asteraceae, Poaceae, Anacardiaceae, and Cucurbitaceae. The first two had 6 genera, each one with 8 and 7 species respectively, whereas the last two had 5 genera each one with 7 and 6 species respectively (Table 2). About 48% of plant families had only one species while 54% of them had only one genus. A list of species, with their utilization and part consumed is presented in Table 3. About 50% of edible plants (57 species) have been cited in more than 50% of surveyed villages (see Table 3).

**Table 2:** **Species and genera richness and relative frequency of edible plant families**

Family	Genera richness	Species richness	Frequency (%)
Asteraceae	6	8	0.07
Poaceae	6	7	0.06
Anacardiaceae	5	7	0.06
Cucurbitaceae	5	6	0.05
Leg-Caesalpinioideae	4	5	0.04
Leg-Papilionoideae	4	5	0.04
Arecaceae	4	4	0.03
Amaranthaceae	2	4	0.03
Solanaceae	2	4	0.03
Tiliaceae	2	4	0.03
Moraceae	1	4	0.03
Bombacaceae	3	3	0.02
Euphorbiaceae	3	3	0.02
Lamiaceae	2	3	0.02
Malvaceae	2	3	0.02
Pedaliaceae	2	3	0.02
Rutaceae	2	3	0.02
Annonaceae	2	2	0.01
Araceae	2	2	0.01
Myrtaceae	2	2	0.01
Rubiaceae	2	2	0.01
Sapindaceae	2	2	0.01
Vitaceae	2	2	0.01
Acanthaceae	1	2	0.01
Apocynaceae	1	2	0.01
Asclepiadaceae	1	1	0.00
Balanitaceae	1	1	0.00
Basellaceae	1	1	0.00
Boraginaceae	1	1	0.00
Bromeliaceae	1	1	0.00
Capparaceae	1	1	0.00
Caricaceae	1	1	0.00
Chrysobalanaceae	1	1	0.00
Clusiaceae	1	1	0.00
Cochlospermaceae	1	1	0.00
Combretaceae	1	1	0.00
Convolvulaceae	1	1	0.00
Cyperaceae	1	1	0.00
Dioscoreaceae	1	1	0.00
Ebenaceae	1	1	0.00
Leg‒Mimosoideae	1	1	0.00
Loganiaceae	1	1	0.00
Meliaceae	1	1	0.00
Moringaceae	1	1	0.00
Musaceae	1	1	0.00
Portulacaceae	1	1	0.00
Sapotaceae	1	1	0.00
Verbenaceae	1	1	0.00

**Table 3:** **Edible plant species collected in two arid (Boukoumbé) and semi-arid (Bassila) areas of Benin**

Voucher specimen code	Local name ^1^	Species	Family	Life form	Phytogeographical zone ^2^	Status ^3^	Utilizations	Parts consumed	Frequency of citations (%)
AS015	Tinoufanti (D), Tinoussanté (M), Maatou (L), Ila (N)	*Abelmoschus esculentus* (L.) Moench	Malvaceae	Herb	SG, S	C	Vegetable	Leaves, Fruits	100
AS016	Moutonmu, Titookanti (D), Kotôlaxa (L), Otché, Osché (N)	*Adansonia digitata* L.	Bombacaceae	Tree	SG, S	UD	Vegetable, Fruit, Condiment, Beverage	Leaves, Pulp, Seed	100
AS017	Munamutimu (D), Yarohu, Waloho, Sonyonma (L), Abo, Arere (N)	*Annona senegalensis* Pers.	Annonaceae	Shrub	SG, S	W	Vegetable, Fruit, Condiment	Flowers, Young leaves, Pulp, Fruits	100
AS018	Tikomaanti, Dikomaanti (D), Nonyrèm, Nèyèrèm (L), Ekpa (N)	*Arachis hypogea* L.	Leg-Papilionoideae	Herb	SG, S	C	Pulse, Vegetable oil	Seeds	100
AS019	Moufodoom (D), Pèèryu (L), Ishin (N)	*Blighia sapida* K.D.Koenig	Sapindaceae	Tree	SG, S	UD	Vegetable, Condiment	Aril, Young leaves	100
AS020	Mufô, Tikonfaati (D), Vèèkou (L), Agougou (N)	*Bombax costatum* Pellegr. & Vuillet	Bombacaceae	Tree	SG, S	UD	Vegetable	Flowers	100
AS021	Mukpiatimu (D), Kparèèyu (L) Agbon (N)	*Borassus aethiopum* Mart.	Arecaceae	Tree	SG, S	W	Fruit, Vegetable	Pulp, Seedling	100
AS022	Mukanbammu (D), Gemburu (L), Ata sisebe, Ata eiye (N)	*Capsicum frutenscens* L.	Solanaceae	Herb	SG, S	C	Vegetable	Fruits	100
AS023	Mukomu (D), Tixoxanté (M), Agougou (N)	*Ceiba pentandra* (L.) Gaertn.	Bombacaceae	Tree	SG, S	W	Vegetable	Young leaves	100
AS024	Tiwadooti (D), Tikpainn'tissêdonté (M), Xonônm (L), Idjabô (N)	*Ceratotheca sesamoides* Endl.	Pedaliaceae	Herb	SG, S	UD	Vegetable	Leaves	100
AS025	Muyua yuam (D), Kpôgolo (N)	*Cissus populnea* Guill. & Perr.	Vitaceae	Liana	SG, S	W	Vegetable, Condiment	Flowers, Young leaves	100
AS026	Tikowounkofanti, Yèkotenko (D), Timoukan’té (M), Mangani (L,N)	*Colocasia esculenta* (L.) Schott	Araceae	Herb	SG, S	C	Vegetable, Tuber	Leaves, Corms	100
AS027	Mumasôkô (D), Iyede (N)	*Detarium microcarpum* Guill. & Perr.	Leg-Caesalpinioideae	Tree	SG, S	W	Fruit	Pulp	100
AS028	Yanwaa (D), Hê (L), Ishu (N)	*Dioscorea cayenensis–D. rotundata* species complex	Dioscoreaceae	Herb	SG, S	C	Tuber	Tubers	100
AS029	Yêpin, Mupiin (D), Gaaya, Gaayu (L), Igi dudu, Karan (N)	*Diospyros mespiliformis* Hochst. ex A.DC.	Ebenaceae	Tree	SG, S	W	Fruit	Pulp	100
AS030	Soja (D, L, N)	*Glycine max* (L.) Merr.	Leg-Papilionoideae	Herb	SG, S	C	Pulse	Grain	100
AS031	Tikwouann’ti (D), Tikonn’té (M), Ankpaman (L), Kpakpala, Amukan (N)	*Hibiscus sabdariffa* L.	Malvaceae	Herb	SG, S	C	Vegetable, Beverage	Leaves, Flowers	100
AS032	Timanuonti (D), Tôxômba (L), Oduku, Adokwin (N)	*Ipomoea batatas* (L.) Lam.	Convolvulaceae	Herb	SG, S	C	Tuber	Tubers	100
AS033	Yapeetaa, Bubaturi (D), Manguna (L), Mangoro (N)	*Mangifera indica* L.	Anacardiaceae	Tree	SG, S	C	Fruit	Fruits	100
AS034	Munuan, Muwassadému (D), Dooso (L), Igba, Ayidan (N)	*Parkia biglobosa* (Jacq.) R.Br. ex G.Don	Leg‒Mimosoideae	Tree	SG, S	W	Fruit, Condiment	Pulp, Seeds	100
AS035	Mukwamkwam (D)	*Sarcocephalus latifolius* (Sm.) E.A.Bruce	Rubiaceae	Shrub	SG, S	W	Fruit	Fruits	100
AS036	Tiwadouanti (D), Tissêdôonté (M), Touhounôm (L), Dossé (N)	*Sesamum radiatum* Schumach. & Thonn.	Pedaliaceae	Herb	SG, S	UD	Vegetable	Leaves	100
AS037	Tiyooti (D), M'la (L), Oka baba, Oka kpikpa (N)	*Sorghum bicolor* (L.) Moench	Poaceae	Herb	SG, S	C	Cereal	Grain	100
AS038	Mupin (D), Ajagbon (N)	*Tamarindus indica* L.	Leg-Caesalpinioideae	Tree	SG, S	W	Fruit, Beverage	Pulp	100
AS039	Tifinhoun'ti (D), Tikoun'téété (M), Elimkpataxa (L), Aroman (N)	*Vernonia amygdalina* Delile	Asteraceae	Shrub	SG, S	C	Vegetable	Leaves	100
AS040	Yakammwà (D), Tura, Tira (L), Ekpa boro (N)	*Vigna subterranea* (L.) Verdc.	Leg-Papilionoideae	Herb	SG, S	C	Pulse	Grain	100
AS041	Titoun'ti (D), Tnainyéritonn’té (M), Tchaassé (L), Ewe, Ewa (N)	*Vigna unguiculata* (L.) Walp.	Leg-Papilionoideae	Herb	SG, S	C	Pulse	Grain	100
AS042	Mutaamu (D), Tambéré (L), Emin (N)	*Vitellaria paradoxa* C.F.Gaertn.	Sapotaceae	Tree	SG, S	UD	Fruit, Vegetable oil	Fruit, Kernel	100
AS043	Tikowounkofanti, Yèkotenko (D), Timoukan’té (M), Mangani (L,N)	*Xanthosoma sagittifolium* (L.) Schott	Araceae	Herb	SG, S	C	Tuber	Tubers	100
AS044	Dimariyo (D), Manzoo (L), Agbado (N)	*Zea mays* L.	Poaceae	Herb	SG, S	C	Cereal	Grain	100
AS045	Tifaanti (D), Tipanoussanté (M), Yôyô (L,N)	*Corchorus olitorius* L.	Tiliaceae	Herb	SG, S	C	Vegetable	Leaves	91.67
AS046	Mukankanwa (D)	*Ficus sycomorus* L.	Moraceae	Tree	SG, S	W	Fruit, Vegetable	Fruits, Young leaves	91.67
AS047	Tikansibouoti (D), Tikli (L)	*Hibiscus asper* Hook.f.	Malvaceae	Herb	SG, S	UD	Vegetable	Leaves, Flowers	91.67
AS048	Yimwa (D), Murii (L), Resi, Iresi (N)	*Oryza sativa* L.	Poaceae	Herb	SG, S	C	Cereal	Grain	91.67
AS049	Mumatonmu, Timantounn’ti (D), Tiwatonn’té (M), Sowarya (L), Akoumanlapka, Ori nla, Osha koro (N)	*Vitex doniana* Sweet	Verbenaceae	Tree	SG, S	W	Vegetable, Fruit	Leaves, Fruits	91.67
AS050	Ifanhanyéi (D), Tixanté (M), Koxolanhoun (L), Ountcho (N)	*Corchorus tridens* L.	Tiliaceae	Herb	SG, S	UD	Vegetable	Leaves	83.33
AS051	Mukpiatikakadata (D)	*Hyphaene thebaica* (L.) Mart.	Arecaceae	Tree	SG, S	W	Fruit	Pulp, Kernel	83.33
AS052	Mussan, Issangnan (D), Jaakpeegna (L), Aku, Asogika, Aso gidoka (N)	*Lannea microcarpa* Engl. & K.Krause	Anacardiaceae	Tree	SG, S	W	Fruit	Pulp	83.33
AS053	Nyakabu (D), Bémeyu (L), Iyeye, Eyeye (N)	*Spondias mombin* L.	Anacardiaceae	Tree	SG, S	W	Fruit	Pulp	83.33
AS054	Ditchéfouwounti, Titchéfouwounti (D), Akaya (N)	*Cleome gynandra* L.	Capparaceae	Herb	SG, S	UD	Vegetable	Leaves	66.67
AS001	Ipoa, Ipordapia, Ipordawan (D), Ipogninimè, Iporni (M)	*Digitaria exilis* (Kippist) Stapf	Poaceae	Herb	S	C	Cereal	Grain	66.67
AS002	Ipoaga (D)	*Digitaria iburua* Stapf	Poaceae	Herb	S	C	Cereal	Grain	66.67
AS055	Munamênii (D)	*Gardenia erubescens* Stapf & Hutch.	Rubiaceae	Shrub	SG, S	W	Fruit, Condiment	Flowers, Fruits, Young leaves	66.67
AS003	Yayomaata (D)	*Pennisetum glaucum* (L.) R.Br.	Poaceae	Herb	S	C	Cereal	Grain	66.67
AS056	Mupotimu (D), Gbaadagnu (L), Goba, Ewé goba (N)	*Psidium guajava* L.	Myrtaceae	Shrub	SG, S	C	Fruit	Fruits	66.67
AS057	Yapeerka (D), Timaati (L), Tomati (N)	*Solanum lycopersicum* L.	Solanaceae	Herb	SG, S	C	Vegetable	Fruits	66.67
AS058	Muboborimu (D), Pempeeya (L), Gogo (N)	*Strychnos spinosa* Lam.	Loganiaceae	Shrub	SG, S	W	Fruit, Vegetable	Fruits, Young leaves	66.67
AS059	Kaju (D), Akadiya (L), Kaju (N)	*Anacardium occidentale* L.	Anacardiaceae	Tree	SG, S	C	Fruit	Kernel	58.33
AS060	Aléfô (D), Bee kumpeeyu (L), Fotètè (N)	*Amaranthus cruentus* L.	Amaranthaceae	Herb	SG, S	C	Vegetable	Leaves	50
AS061	Muporicoetimu (D)	*Balanites aegyptiaca* (L.) Delile	Balanitaceae	Tree	SG, S	C	Fruit	Fruits	50
AS062	Tinonyawouti (D), Tipékênonté (M), Nyaayu (L), Tchôkôyôkôtô (N)	*Celosia argentea* L.	Amaranthaceae	Herb	SG, S	C	Vegetable	Leaves	50
AS063	Mutaarmu (D), Agusi, Teneyu (L), Kaka, Egusi (N)	*Lagenaria siceraria* (Molina) Standl.	Cucurbitaceae	Herb	SG, S	C	Vegetable	Fruits, Seeds	50
AS064	Tifôônouwôti (D), Agbédéxatou (L), Kpaki (N)	*Manihot esculenta* Crantz	Euphorbiaceae	Shrub	SG, S	C	Root	Roots	50
AS065	Tibòdayati, Tibòsèyenti (D), Tignainté (M), Assôou (L), Alounmamba, Aribala (N)	*Ocimum gratissimum* L.	Lamiaceae	Herb	SG, S	C	Spice	Leaves	50
AS004	Muganyan, Mounannikmon (D), Ubamingbu (M)	*Sclerocarya birrea* (A.Rich) Hochst.	Anacardiaceae	Tree	S	W	Fruit, Beverage	Fruits, Pulp	50
AS066	Muwaadonmu (D), Saamu, Nareer (L), Yonmonti, Nyamoti, Ewe ekutu (N)	*Sesamum indicum* L.	Pedaliaceae	Herb	SG, S	C	Vegetable	Leaves, Seeds	50
AS067	Tikawounfanti (D), Tikann’té (M), Gboma (L), Gboma, Kpatakpakô (N)	*Solanum macrocarpon* L.	Solanaceae	Herb	SG, S	C	Vegetable	Leaves	50
AS068	Demumuda daniira (D), Akutongnu, Afotongnu (L), Orombo didu, Orombo igun (N)	*Citrus sinensis* (L.) Osbeck	Rutaceae	Tree	SG, S	C	Fruit	Fruits	41.67
AS005	Mumasôkô (D), Ogbôgbô (N)	*Detarium senegalense* J.F.Gmel.	Leg-Caesalpinioideae	Tree	S	W	Fruit	Pulp	41.67
AS069	Mupénuamu (D)	*Ficus asperifolia* Miq.	Moraceae	Shrub	SG, S	W	Fruit	Fruits	41.67
AS070	Tinoukounti (D), Tilétoussi (L), Djagou-djagou (N)	*Justicia tenella* (Nees) T.Anderson	Acanthaceae	Herb	SG, S	UD	Vegetable	Leaves, Shoot, Stem	41.67
AS071	Tikoun'tééti (D), Arikoro (N)	*Vernonia colorata* (Willd.) Drake	Asteraceae	Shrub	SG, S	W	Vegetable	Leaves	41.67
AS072	Tipébouoti (D), Oubouonou, Ibouoni (M)	*Acmella oleracea* (L.) R.K.Jansen	Asteraceae	Herb	SG, S	UD	Vegetable	Leaves	33.33
AS073	Yapeertora (D), Ibekpe, Igi bekpe (N)	*Carica papaya* L.	Caricaceae	Tree	SG, S	C	Fruit	Fruits	33.33
AS083	Tinacanti (D), Egusi, Itoô (N)	*Citrullus mucosospermus* Fursa	Cucurbitaceae	Herb	SG	C	Vegetable	Seeds	33.33
AS084	Gbolo (N)	*Crassocephalum crepidioides* (Benth.) S.Moore	Asteraceae	Herb	SG	UD	Vegetable	Leaves	33.33
AS085	Mupomu (D), Be yiya (L), Igi okpe, Okpe (N)	*Elaeis guineensis* Jacq.	Arecaceae	Tree	SG	C	Vegetable oil	Pulp	33.33
AS006	Munapuo (D)	*Ficus dicranostyla* Mildbr.	Moraceae	Shrub	S	W	Fruit, Vegetable	Fruits, Young leaves	33.33
AS074	Mupémi (D)	*Lannea acida* A.Rich.	Anacardiaceae	Tree	SG, S	W	Fruit	Pulp	33.33
AS086	Odôdô (N)	*Launaea taraxacifolia* (Willd.) Amin ex C.Jeffrey	Asteraceae	Herb	SG	W	Vegetable	Leaves	33.33
AS075	Mounpêkom (D), Kêpiénouakê (M), Agdêdêxatou, Lôtaxa (L), Ekégnibo (N)	*Moringa oleifera* Lam.	Moringaceae	Shrub	SG, S	C	Vegetable	Leaves, Seeds	33.33
AS076	Yèkodiyè (D), Kainton’ko (M), Kouwoundou (L), Tchidifulè, Yèbè, Iman (N)	*Solanum aethiopicum* L.	Solanaceae	Herb	SG, S	C	Vegetable	Immature fruits, Young leaves	33.33
AS077	Yêmontouo (D), Kamplékankann’dê (L), Odondon, Gbure, Gure (N)	*Talinum triangulare* (Jacq.) Willd.	Portulacaceae	Herb	SG	UD	Vegetable	Leaves	33.33
AS087	Faso (D), Saada kuriji (L), Yraha, Eruju (N)	*Uvaria chamae* P.beauv.	Annonaceae	Shrub	SG	W	Fruit	Pulp	33.33
AS088	Mubuo (D), Igi ata (N)	*Zanthoxylum zanthoxyloides* (Lam.) Zepern. & Timber	Rutaceae	Shrub	SG	W	Vegetable, Spice	Leaves, Root bark	33.33
AS078	Yépètum (D), Paltiyu (L), Otili (N)	*Cajanus cajan* (L.) Millsp.	Leg-Papilionoideae	Shrub	SG, S	C	Pulse	Seeds	25
AS089	Tipeti (D), Elegede (N)	*Cucurbita maxima* Duchesne	Cucurbitaceae	Herb	SG	C	Vegetable	Fruits, Leaves	25
AS090	Anwin, Iwin (N)	*Dialium guineense* Willd.	Leg-Caesalpinioideae	Tree	SG	W	Fruit	Pulp	25
AS007	Mukankanwamimu (D)	*Ficus sur* Forssk.	Moraceae	Tree	S	W	Fruit	Fruits	25
AS091	Moussannoum, Mussantiwamu (D)	*Grewia mollis* Juss.	Tiliaceae	Shrub	SG	W	Vegetable, Condiment	Flowers, Fruits, Leaves	25
AS008	Issian (D)	*Lannea barteri* (Oliv.) Engl.	Anacardiaceae	Tree	S	W	Fruit	Pulp	25
AS092	Aminagnu (L), Ogede (N)	*Musa* sp.	Musaceae	Herb	SG	C	Fruit	Fruits	25
AS093	_	*Neocarya macrophylla* (Sabine) Prance	Chrysobalanaceae	Shrub	SG	W	Fruit	Kernel, Pulp	25
AS009	Mupeketatié (D)	*Paullinia pinnata* L.	Sapindaceae	Liana	S	W	Fruit	Pulp	25
AS094	Kodjonou (L), Ekuso (N)	*Pentadesma butyracea* Sabine	Clusiaceae	Tree	SG	W	Vegetable oil	Kernel	25
AS095	Tètè dudu wèrè (N)	*Amaranthus viridis* L.	Amaranthaceae	Herb	SG	W	Vegetable	Leaves	16.67
AS010	Tawotatchoyan (D)	*Antidesma venosum* Tul.	Euphorbiaceae	Shrub	S	W	Fruit	Fruits	16.67
AS096	Egusi (L), Itoô (N)	*Cucumeropsis mannii* Naudin	Cucurbitaceae	Herb	SG	C	Vegetable	Seeds	16.67
AS079	Yanaacemmora (D), Ofio, Omu, Amu (N)	*Cyperus esculentus* L.	Cyperaceae	Herb	SG, S	C	Tuber	Tubers	16.67
AS097	Suyouxo (L), Ogbé akuko (N)	*Heliotropium indicum* L.	Boraginaceae	Herb	SG	W	Vegetable	Leaves	16.67
AS098	Djagou-djagou (N)	*Justicia insularis* T.Anderson	Acanthaceae	Herb	SG	UD	Vegetable	Leaves	16.67
AS080	Koupanouwôkou (D), Akohoun (N)	*Ocimum basilicum* L.	Lamiaceae	Herb	SG, S	W	Vegetable, Spice	Leaves	16.67
AS099	Mukpétida (D)	*Phoenix reclinata* Jacq.	Arecaceae	Tree	SG	W	Fruit	Fruits	16.67
AS100	Ibo gidi (N)	*Saba comorensis* (Bojer) Pichon	Apocynaceae	Liana	SG	W	Fruit	Pulp	16.67
AS101	Ibo gidi (N)	*Saba senegalensis* (A.DC.) Pichon	Apocynaceae	Liana	SG	W	Fruit, Beverage	Pulp	16.67
AS081	Mucotamu (D), Isenren, Sansan boto, Adere (N)	*Syzygium guineense* (Willd.) DC.	Myrtaceae	Shrub	SG, S	W	Fruit	Pulp	16.67
AS011	Dipugedi (D)	*Trichilia emetica* Vahl	Meliaceae	Tree	S	W	Fruit	Aril	16.67
AS102	Ekunhun ahun (N)	*Ananas comosus* (L.) Merr.	Bromeliaceae	Herb	SG	C	Fruit	Fruits	8.33
AS103	Agni (N)	*Anogeissus leiocarpa* (DC.) Guill. & Perr.	Combretaceae	Tree	SG	W	Vegetable	Young leaves	8.33
AS104	Gbogboloki (N)	*Basella alba* L.	Basellaceae	Herb	SG	UD	Vegetable	Leaves, Shoot	8.33
AS105	Asha (N)	*Bridelia ferruginea* Benth.	Euphorbiaceae	Shrub	SG	W	Food processing	Bark	8.33
AS012	Kunaakoobu (D)	*Calotropis procera* (Aiton) R.Br.	Asclepiadaceae	Shrub	S	UD	Food processing	Leaves	8.33
AS106	Adjèmanwofô (N)	*Celosia trigyna* L.	Amaranthaceae	Herb	SG	W	Vegetable	Leaves	8.33
AS107	Natataka (L)	*Chrysanthellum indicum* DC.	Asteraceae	Herb	SG	W	Vegetable	Leaves	8.33
AS082	Demmuda dadaara (D), Osan orombo, Osan wewe (N)	*Citrus limon* (L.) Burm.f.	Rutaceae	Tree	SG, S	C	Fruit	Fruits	8.33
AS108	Omronlugboko (N)	*Cochlospermum planchonii* Hook.f.	Cochlospermaceae	Shrub	SG	W	Condiment	Rootstock	8.33
AS109	Gbolo (N)	*Crassocephalum rubens* (Juss. ex Jacq.) S.Moore	Asteraceae	Herb	SG	UD	Vegetable	Leaves	8.33
AS110	Kanmblê (L)	*Cucurbita moschata* Duchesne	Cucurbitaceae	Herb	SG	C	Vegetable	Fruits	8.33
AS013	Timammuti (D)	*Cymbopogon giganteus* Chiov.	Poaceae	Herb	S	W	Spice	Leaves	8.33
AS111	Tiyankwoun’ti (D), Gnainrissé angbaman (L)	*Cyphostemma adenocaule* (Steud. ex A.Rich) Wild & R.B.Drumm.	Vitaceae	Herb	SG	W	Vegetable	Leaves	8.33
AS112	Odundun odo (N)	*Emilia sonchifolia* (L.) DC. ex Wight	Asteraceae	Herb	SG	W	Vegetable	Leaves	8.33
AS014	Mussantipê (D)	*Grewia lasiodiscus* K.Schum.	Tiliaceae	Shrub	S	W	Condiment	Fruits	8.33
AS113	Anikan gbiju (N)	*Hoslundia opposita* Vahl	Lamiaceae	Shrub	SG	W	Vegetable, Spice	Leaves	8.33
AS114	Ejinrin (N)	*Momordica charantia* L.	Cucurbitaceae	Herb	SG	W	Vegetable	Leaves	8.33
AS115	Adjan'gulu (N)	*Senna occidentalis* (L.) Link	Leg-Caesalpinioideae	Herb	SG	W	Vegetable	Leaves	8.33

^1^D = Ditamari, M = Mbermé, L = Lokpa, N = Nagot; ^2^S = Sudanian zone, SG = Sudano-Guinean zone; ^3^C = Cultivated, UD = Under domestication, W = Wild.

Estimation of cultivated species richness indicated 45 species with Confidence Interval (CI) of 42 to 48 species belonging to 23 families and 38 genera. They are dominated by vegetable (47%) species (e.g. *Abelmoschus esculentus*, *Corchorus olitorius*, *Solanum macrocarpon*, *Vernonia amygdalina*) and fruit (20%) species (e.g. *Citrus sinensis*, *Mangifera indica*, *Psidium guajava*). Cereals (e.g. *Oryza sativa*, *Sorghum bicolor*, *Zea mays*) and pulses (e.g. *Arachis hypogea*, *Glycine max*, *Vigna unguiculata*) accounted for 24% of cultivated species and roots and tubers for 13% (e.g. *Colocasia esculenta*, *Dioscorea cayenensis–rotundata* complex, *Ipomoea batatas*, *Manihot esculenta*).

The estimated wild edible species richness indicated 70 species (CI: 66–76) representing 61% of edible plants collected. They also represent 46% of the most cited species (species cited in more than 50% of surveyed villages) by communities (Table 3). About 25% of them are under domestication and had dual status depending on the village. Some species are entirely wild and collected only when needed (e.g., *Ceratotheca sesamoides*, *Cissus populnea*), others are maintained in agricultural environments (fields and fallow) or around habitats (e.g., *Adansonia digitata*, *Vitellaria paradoxa*, *Parkia biglobosa*, *Blighia sapida*) and others have dual status depending on the village (e.g., *Sesamum radiatum*, *Hibiscus asper*, *Justicia tenella*, *Corchorus tridens, Talinum triangulare*). Wild edible species belong to 38 families and 61 genera. Vegetable species represent about 57% while fruit species account for roughly 47% of wild edible plants collected. Several life forms were recorded, but dominated by trees (36%) and herbaceous species (33%). Many wild species (41%) have multiple edible parts, including edible leaves, fruits, flowers, pulps, kernel, and/or seeds (e.g. *Adansonia digitata*, *Annona senegalensis*, *Blighia sapida*, *Cissus populnea*). About 96% of herbs are used as vegetables whereas about 80% of tree species provide fresh fruits (Table 4). Shrubs provide fruits (56%) and also used as vegetable (39%). Other uses (about 50%) were recorded for shrubs and included purgative and laxative (e.g. *Tamarindus indica*), stimulation of milk production in lactating women (e.g. *Zanthoxylum zanthoxyloides*), toothbrush (e.g. *Vernonia amygdalina*). Comparison of estimated richness between wild and cultivated species indicated that wild plant richness is significantly higher than cultivated species richness (Figure 2a, p < 0.001).

**Table 4:** **Wild species richness and percentages (in brackets) of use types per life form**

Species counts		Uses		
		Vegetables	Fruits	Other uses
Life forms				
Herbaceous	23	22 (96%)	0 (0%)	2 (9%)
Liana	4	1 (25%)	3 (75%)	2 (50%)
Shrub	18	7 (39%)	10 (56%)	9 (50%)
Tree	25	9 (36%)	20 (80%)	7 (28%)
Counts by use type	70	39 (56%)	33 (47%)	20 (29%)

**Figure 3:**
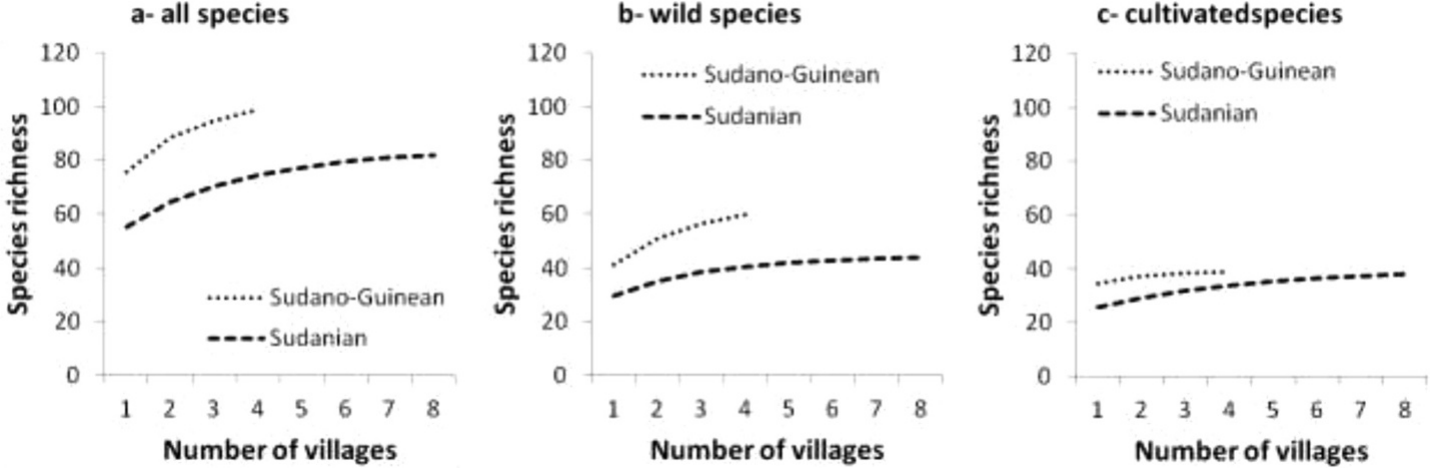
**Estimated edible food plant species richness (a-all species, b-wild species and c-cultivated species) between two arid (Boukoumbé) and semi-arid (Bassila) areas of Benin based on incidence data.**

The estimation of species richness per region indicated 99 species (CI: 94–104) with about 60% of wild species (59 species) in Bassila and 82 species (CI: 79–89) with about 54% of wild species (44 species) in Boukoumbé. Overall, edible species richness in Bassila was significantly higher than in Boukombé (Figure 3a, p < 0.05). The same trend was observed when taking into account only wild species (Figure 3b, p < 0.01). However, there is no significant difference between the two areas when considering only cultivated species (Figure 3c, p = 0.1334). Thirty five out of 45 cultivated species collected are shared by the two areas (e.g. *Zea mays*, *Sorghum bicolour*, *Vigna unguiculata*, *Arachis hypogea*, *D. cayenensis–rotundata* complex, *Manihot esculenta*, *Abelmoschus esculentus*, *Corchorus olitorius*, *Mangifera indica*, *Psidium guajava*). Main species only cultivated in Bassila include *Ananas comosus* and *Elaeis guineensis. Digitaria exilis*, D. *iburua* and *Pennisetum glaucum* are the three species only found in Boukoumbé.

**Figure 4:**
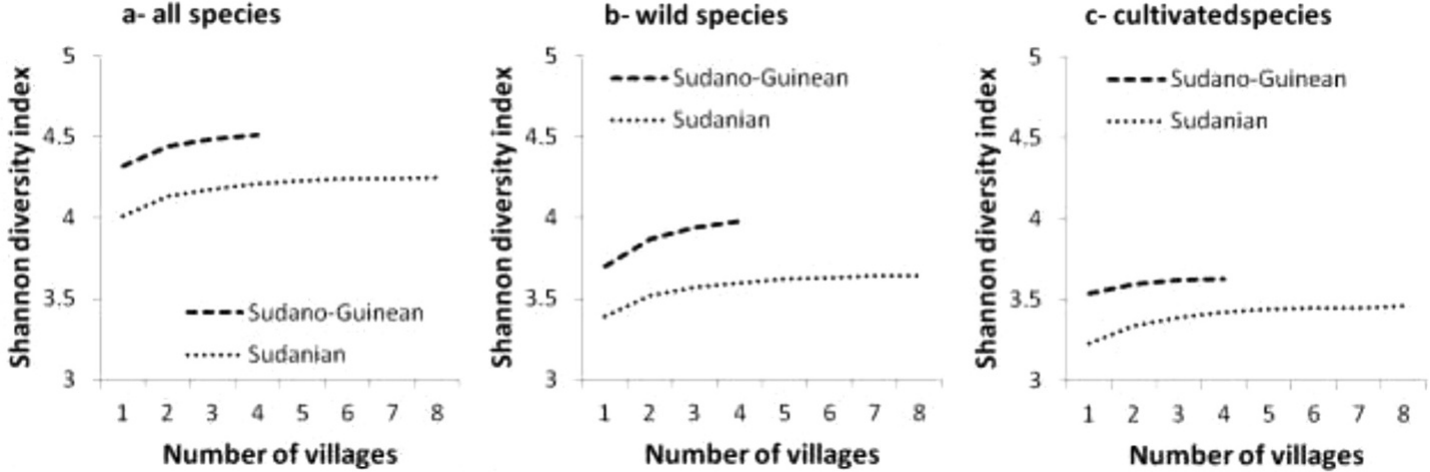
**Estimated Shannon diversity index for edible food plant species (a-all species, b-wild species and c-cultivated species) between two arid (Boukoumbé) and semi-arid (Bassila) areas of Benin based on incidence data.**

The Shannon-Wiener diversity index was high, up to 4.5 for all categories and followed an asymptotic function against the sample frequency (Figure 2b). Wild species diversity is significantly higher than cultivated species diversity (Figure 2b, p < 0.001). The Shannon-Wiener diversity index was equally high in the two regions. It was close to 4.5 and 4.2 in Bassila and Boukoumbé respectively. Overall, edible species diversity in the semi-arid Sudano-Guinean area was significantly higher than in the arid Sudanian area (Figure 4a, p < 0.001). The same trend is observed when taking into account only wild species (Figure 4b, p < 0.001) and also cultivated species only (Figure 4c, p < 0.001).

**Figure 5:**
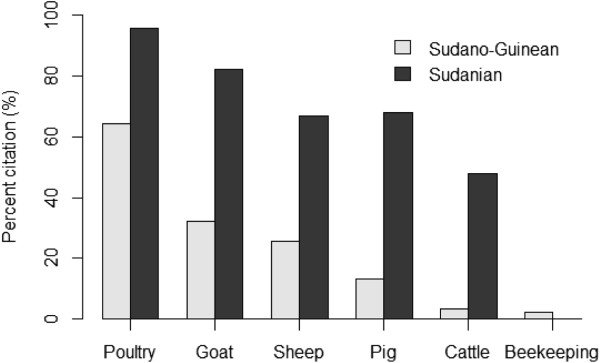
**Animal species raised in two arid (Boukoumbé) and semi-arid (Bassila) areas of Benin.**

Although about 83% of respondents indicated that they had livestock, livestock breeding differed significantly between the two areas. More farmers were involved in livestock breeding in arid area than in semi-arid one (Figure 5, p < 0.001). The majority of them explained that livestock resources are used for household needs (e.g., consumption, cultural ceremonies) and commercialized to earn additional financial resources. They argued that this supplementary resource is crucial not only during food shortage period but also during agricultural work period. Animal species raised were dominated by poultry and small ruminants (Figure 5).

**Figure 6:**
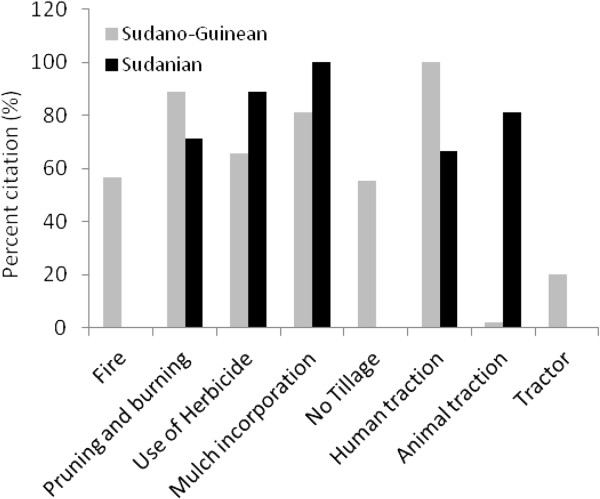
**Land clearing and preparation practices in two arid (Boukoumbé) and semi-arid (Bassila) areas of Benin.**

**Figure 7:**
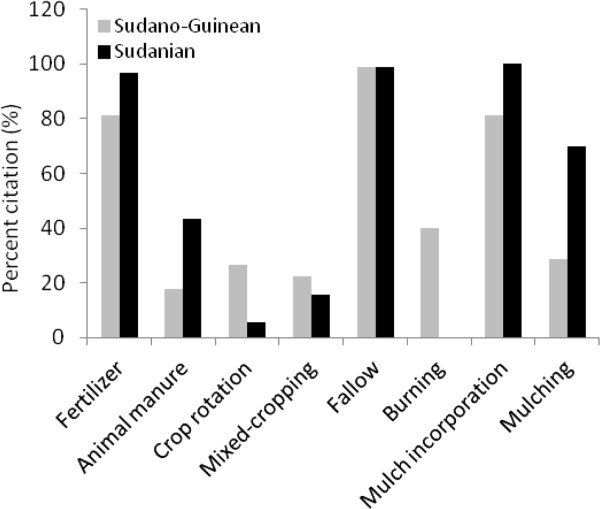
**Soil fertility management practices in two arid (Boukoumbé) and semi-arid (Bassila) areas of Benin.**

**Figure 8:**
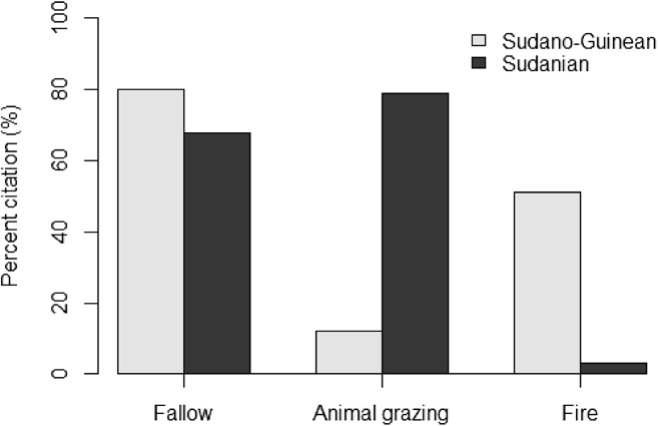
**Field management after harvest practices in two arid (Boukoumbé) and semi-arid (Bassila) of Benin.**

**Figure 9:**
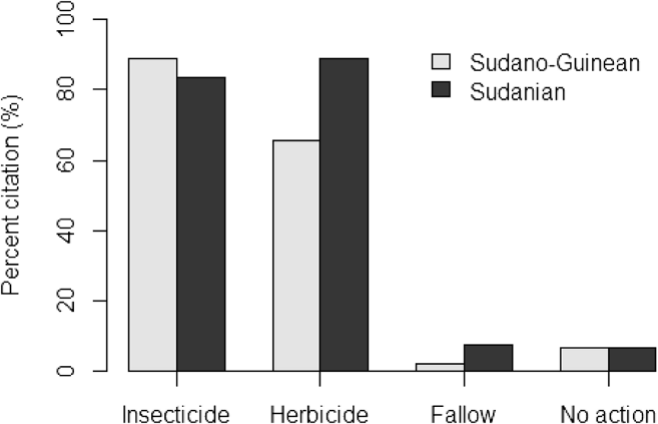
**Pest management practices in two arid (Boukoumbé) and semi-arid (Bassila) areas of Benin.**

Although farmers in Bassila exploit an overall higher edible plant diversity compared to their counterpart in Boukoumbé, this is not necessary translated into resource-conservation practices. Land clearing and land preparation practices developed were region-dependent (Figure 6, p < 0.001). Farmers in the semi-arid area used fire to clear new land. On old fields seedling and sapling are pruned and burnt. They also used human traction and to some extent tractors. We noticed no tillage. In the drier area however, the majority of farmers indicated that they use herbicide to clear land. Animal traction is used for tillage and mulches are systematically incorporated during the tillage as soils are degraded. Soil fertility management practices were also region-dependent (Figure 7, p < 0.001). Chemical fertilizer, animal manure, mulch incorporation and mulching were more used by farmers in the arid area while farmers in the semi-arid area still relied on slash-and-burn cultivation, crop rotation, burning of crop residue and mulches, and mixed-cropping. Similarly, field management practices after harvest were related to region where farmers were living (Figure 8, p < 0.001). Farmers in the semi-arid area left fields for fallow and/or burnt crop residues. They explained that burning crop residues after harvest reduces weed pressure at beginning of the next cropping season. It also avoids attracting the nomadic Fulani people and their herds into their fields because cattle increase soil degradation and make tillage labour difficult at the next cropping season. However, farmers in the arid area left fields for fallow and/or grazed their own cattle and small ruminants on the fields. They explained that crop residues serve as fodder for their livestock and the manure serves as fertilizer to crops. Contrary to others farming practices, there is no significant difference between the two municipalities regarding pest management practices (Figure 9, p = 0.1974). Farmers widely used chemical insecticide in both regions, mainly on cotton and to some extent on cowpea.

### Relationship among communities in term of choice of edible plants

**Figure 10:**
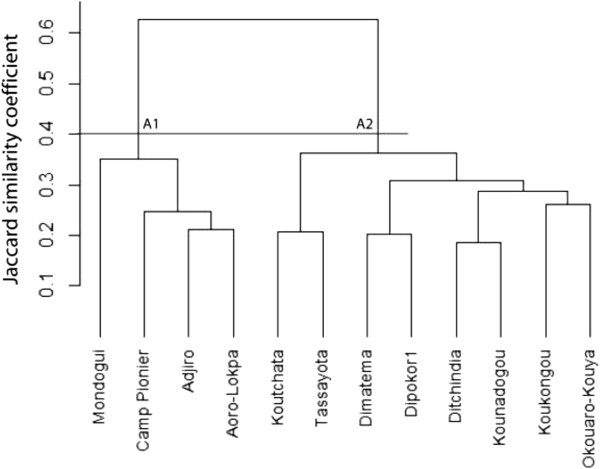
**Dendrogram showing the similarity among sociolinguistic groups based on all edible plant species in two arid (Boukoumbé) and semi-arid (Bassila) areas of Benin.**

**Figure 11:**
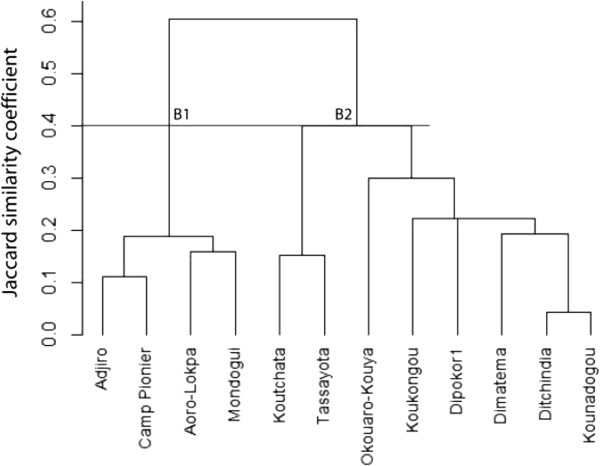
**Dendrogram showing the similarity among sociolinguistic groups based on cultivated species in two arid (Boukoumbé) and semi-arid (Bassila) areas of Benin.**

**Figure 12:**
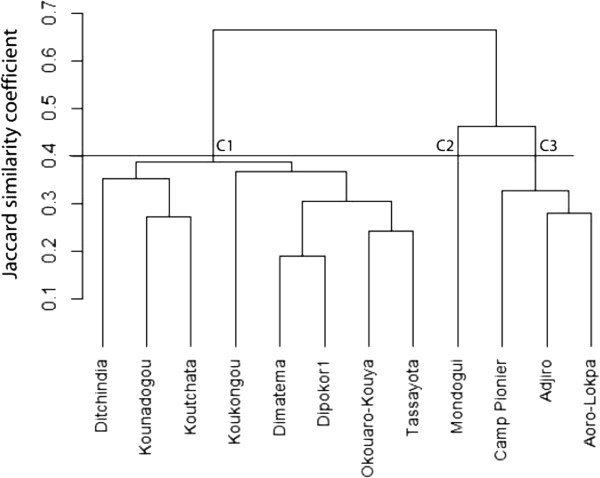
**Dendrogram showing the similarity among sociolinguistic groups based on wild species in two arid (Boukoumbé) and semi-arid (Bassila) areas of Benin.**

To understand the impact of agroecological context and socio-cultural attributes on edible plants selection by a community, we performed a cluster analysis using villages as operational units (Figures 10, 11 and 12). Each village is dominated by only one sociolinguistic group. The dendrogram in Figure 10 presents data on all edible plants together. At 40% of similarity, it shows two groups that revealed the two phytogeographical zones. Cluster A1 is composed of all villages of Bassila and no clear grouping according to sociolinguistic membership was noted within the cluster. Cluster A2 is composed of all villages of Boukoumbé. There is no clear grouping according to sociolinguistic membership too. The dendrogram in Figure 11 is based solely on cultivated species, and shows at 40% of similarity two groups based on phytogeographical zones. Cluster B1 is composed of all villages of Sudano-Guinean municipality and Cluster B2 of all villages of Sudanian municipality. There is no clustering according to sociolinguistic membership. The data in Figure 12 take into account wild species only, the same trend was observed although this topology revealed three clusters: Cluster C1 composed of all villages of Sudanian region, Cluster C2 composed of one village (Modogui, a Nagot socio-linguistic community) of Sudano-Guinean region and Cluster C3 of the rest of villages (which are of immigrant community) of Sudano-Guinean region.

## Discussion

To the best of our knowledge, this is the first empirical study in Benin as well as in West Africa comparing the diversity of edible plants used by communities in two contrasting areas (in terms of ecological and socioeconomic characteristics). Similarly, exploring how communities produce their crops (farming practices’ analysis) combined with what they used as food plants (edible food plants diversity analysis) give new insights on local people food basket.

The total number of 115 species consumed as food plants in the two municipalities is considerable. It represents about 4% of the floristic diversity of the whole country [33]. Among the bulk of food plants used, wild species represent 61% (70 species) and 46% of the most cited species by communities. The number of wild edible plants used in the two municipalities represents 43% of the total number of non-wood edible forest plant resources collected during a country-wide market survey in Benin [52] and is lower than the diversity of noncultivated plant species (87 species) collected in the southwest Benin and the southeast Togo [53]. Vegetable species represent about 57% while fruit species account for roughly 47% of wild edible plants collected. This highlights the potential role of wild vegetables and fruits in improving food and nutritional security of rural communities [54–56]. Wild species provide various food products for household’s daily diets, especially for poorer households in northern Benin [40]. Our results indicate that in dry areas wild plants still constitute an important asset in addressing food security by ensuring the availability and accessibility of food plants. Their contribution to food security in rainforest regions has also been demonstrated [57, 58]. According to Bharucha and Pretty [59] wild plants and animals continue to form a significant proportion of the global food basket, and their roles and values in agricultural systems may be set to grow as pressures on agricultural productivity increase. Based on our results, we emphasize the important value of wild edible resources in food consumption in dry areas. Their roles in farmers’ global livelihoods are likely to be more important, as reported by many authors [22, 29, 40, 57, 60–62]. The improvement of food security in developing countries will depend on the placement of wild edible resources in agricultural policies as well. A thorough documentation of wild edible plants and their contribution to household diet will help improve knowledge on the under-valued biological and cultural diversity that are of importance to address food security, environmental and economic sustainability [63]. Moreover, a diversification with greater use of highly valuable but presently under-utilized crops and species should be an essential element of any model for sustainable smallholder agriculture [4].

About 25% of wild edible plants are under domestication and can be found in one or other step in the “bringing into cultivation” phase of the plant domestication process described by Vodouhè and Dansi [64]. Some species are entirely wild and collected only when needed, others are maintained in agricultural environments (fields and fallow) or around habitats and others have dual status depending on the village. Asteraceae and Cucurbitaceae families, which are among the most important plant families, also showed a high vegetable species richness in Benin and Togo [24, 30]. These findings reveal the importance of these species and botanical families to farmers and to food diets as well. Indeed, food uses is the main reason that motivates local communities in Benin for plant domestication [64]. Therefore, there is a need to consolidate the on-going researches on domestication process of some of species such as *S. radiatum*, *J. tenella*, *A. digitata*, *V. doniana*, *C. rubens* and *C. crepidioides* [24, 60, 65–71] to ensure and enhance the availability, accessibility, and utilization food plants. Moreover, the promotion of the ‘’bringing into cultivation” practices contributes to not only plant domestication but also to promoting diversity, increasing its sustainable utilization and conservation of agrobiodiversity *in situ* [64].

Trees, shrubs and lianas account for about 70% of wild edible plants collected. Many of them are maintained in agricultural environment forming the so called “agroforestry parklands”, a widespread traditional land use system in West African dry savannas in which trees and shrubs are intentionally spared and let scattered on farmlands and fallows [72]. Indeed, agroforestry systems offer a number of ecosystem services and environmental benefits, including soil fertility improvement, soil and water conservation, and environmental protection by maintaining ecological stability and conservation of biodiversity [73]. These ecosystem functions have been at the centre of the local ecological knowledge guiding the management options of the farmers [74]. As evidenced by Assogbadjo *et al.* [75] wild edible species retained in agroforestry systems in Benin depends on farmers’ knowledge on species contribution to food, its use in traditional medicine and ceremonies and farmers’ perception of its availability in natural vegetation. By regulating ecosystem functions such as nutrient recycling, water use, species diversity and agrochemical pollution agroforestry can sustain agricultural intensification and food security in Africa [76, 77]. Moreover, agroforestry systems promote integrated management systems that relate livelihoods and ecosystem service functions to agricultural production and is therefore often considered as a way to sustainably intensify farming practices for enhanced food security, using socially and cost-effective management techniques [76, 78]. Thus, the improvement of agroforestry practices will benefit wild edible plants and enhance the role of these systems in biodiversity conservation and food provision.

Our study revealed that edible species richness and diversity declines from the semi-arid to the arid zone. Moreover, the diversity of wild edible plants used in Bassila (59 species) is higher than in the buffer zone of the Lama forest in southern Benin (48 botanically identified plants) [58]. It is also higher than in other localities in the Sudano-Guinean region (41 wild edible plants in the buffer zone of the Dan forest in Djidja District [60] and 40 wild edible plants in the Collines region in central Benin [21]). These findings are consistent with results from Achigan-Dako *et al.* [24] and Salako *et al.* [79] in Benin on traditional vegetable and home gardens species respectively. This trend could be explained by the fact that in West Africa diversity tends to decline with declining precipitation and from south to north [80]. This south–north climatic gradient which results from the combining effects of annual rainfall, length and severity of the dry season, and air humidity, the soils and geological factors are the major environmental factors underlying plant diversity patterns [81]. Other socio-cultural drivers may also explain this situation. Indeed, the Sudano-Guinean region is a transitional zone between the humid Guinean region in the south and the drier Sudanian region in the north and is a receptacle of strong dynamic migration [23]. The transitional attribute combined with cultural exchange and population migrations within the region could contribute to the observed greater edible species richness and diversity. Cultivated species richness and composition did not significantly differ between the semi-arid and the arid areas. Therefore, the overall difference observed in species richness among the two regions is attributed to wild species. However, as evidenced by Mulumba *et al.* [82], the use of crop varietal diversity is a risk-minimizing strategy, we speculate that varietal diversity and richness used might be higher in the arid Sudanian zone.

Achieving food security in arid and semi-arid regions of Benin requires productive agriculture, and the wide range of edible plant diversity and farming practices [2] should not be overlooked. A comparative analysis of farming system between the two regions indicated that farming practices were significantly related to phytogeographical regions except for pest management practices. Slash-and-burn is still ongoing in semi-arid Sudano-Guinean area as environmental conditions (e.g. precipitation, soil, plant diversity) are more favourable. Fire and burning were heavily used in land preparation, soil fertility management and field management after harvest. New land is converted each year to agriculture, reducing forests and savannas. Cotton expansion is a main driver of forests, savannas and land degradation resulting in loss of biodiversity in West African savannas [83–85]. In the arid Sudanian area farmers have developed more resource-conserving practices as a consequence of land degradation and climatic constraints that they face. They integrate more livestock resources to meet their livelihood needs. As a result they use animal manure for soil fertility management, animal traction for tillage, and graze their livestock on their fields after harvest. We recommended that extension services take into account differences between the two areas and provide farmers with insights and technologies on appropriate crops based on land and resources availability. At the same time, account should be given to intensive awareness-raising about best farming practices. Sustainable or resource-conserving farming practices developed by farmers in less favourable areas need to be strengthened and supported, and also promoted in more favourable areas so as to preserve agroecosystem and natural resources for upcoming generations. Indeed, appropriate agricultural management practices are critical to realizing the benefits of ecosystem services and reducing disservices from agricultural activities [86]. Moreover, the strengthening of livestock-crop integration is crucial for ecological intensification of agriculture to achieve current and future food security and environmental sustainability [87].

Many findings highlighted the importance of socio-cultural attributes in the utilization and values that a community gives to plant resources [14–16, 18–22]. This is illustrated in Figures 10 and 12, where socio-cultural attributes are indicated to play an important role in plant use. For instance, in Cluster A1 Modogui, a Nagot sociolinguistic group village stands alone; Aoro-Lokpa and Adjiro, two Lokpa villages, are grouped into the same sub-cluster while Camp pionier, a Ditamari sociolinguistic group stands alone. Cluster A2 is composed of Ditamari and M’Bermé, two ethnic groups that have high linguistic and cultural affinities. These two ethnic groups formed with other minor ethnic groups the Otamari linguistic group. Ditamari (in general term Otamari group) and Lokpa from the Sudanian region are two ethnic groups that are the principal actors of migratory dynamics in Benin [23, 36]. Members of these groups are motivated to leave their homes in Atacora because of soil degradation, in search of the virgin and fertile lands [23, 36, 37]. A more clear trend is observed when considering solely wild species, where Nagot ethnic group stands alone in one cluster, immigrant ethnic groups stand alone in Sudano-Guinean zone and Otamari linguistic group stand alone too. However, socio-cultural attributes are not sufficient to explain trends revealed by the dendrograms. Villages were mainly clustered according to phytogeographical regions. Camp pionier, a Ditamari village in Sudano-Guinean, does not group with others Ditamari villages in Sudanian region. Bio-geographical factors also play a role in the choice of edible plants by community as also demonstrated for traditional vegetables [24] and for yams’ varietal diversity [23] in Benin. We conclude that there is a complementarity between socio-cultural attributes of community and bio-geographical factors that may explain the choice of edible food plants, especially for wild species. This conclusion is consistent with previous results that had shown that the knowledge and consumption of wild edible plants follows a pattern according to ecological conditions of the gathering environments, as well as the cultural heritage of the communities [24, 88]. Our results also indicated that when a community moved to a more favourable area (e.g. precipitation, soil, plant diversity), members adapted their choice to the plant resources available. Other variables such as distance to markets and urbanization may also affect the choice of edible plants. But, here we are in a context of smallholders farming, and agricultural activities are mainly oriented towards households’ subsistence. Distance to markets and urbanization were not tested in our study areas which are rural settings.

## Conclusions

This study revealed the diversity and richness of edible foods plants and analyzed farming practices in arid and semi-arid areas in Benin. We found that wild species play an important role in food consumption of communities in dry areas, and that the diversity of edible plants is higher in the semi-arid area than in the arid one. However, farmers in the less favourable arid area developed advanced resource-conserving practices compared to their counterparts of the favourable semi-arid area. We conclude that if food security has to be addressed, the production and consumption policies must be re-oriented toward the recognition of the place of wild edible plants and farm management practices developed by farmers. For this to happen we suggest a number of policy and strategic decisions as well as research and development actions, including: (1) intensive awareness raising on best farming practices; (2) thorough documentation of wild edible plants and their contribution to household diet; (3) promotion of the ‘’bringing into cultivation” practices; (4) strengthening of livestock-crop integration; and (5) the improvement of agroforestry systems.

There is a room for further investigations on how climatic gradients shape the utilization patterns of crop varietal diversity. This will help develop a sound on-farm conservation approach of plant genetic resources. Moreover, since the frequency of consumption of each species was not documented, more investigations with adapted methodology are therefore required to better understand the importance of each species in the diets. Twenty-Four Hour Recall method (see [58]) is a useful approach that can help gather information regarding diets and nutritional habits.
